# Supplementary material for: A benchmark of optimization solvers for genome-scale metabolic modeling of organisms and communities
Source: mSystems. 2024 Jan 22;9(2):e00833-23. doi: 10.1128/msystems.00833-23 (PMC10878033; doi:10.1128/msystems.00833-23)
Supplement: Fig. S1 — Benchmark using pFBA simulation. [file msystems.00833-23-s0001.docx]

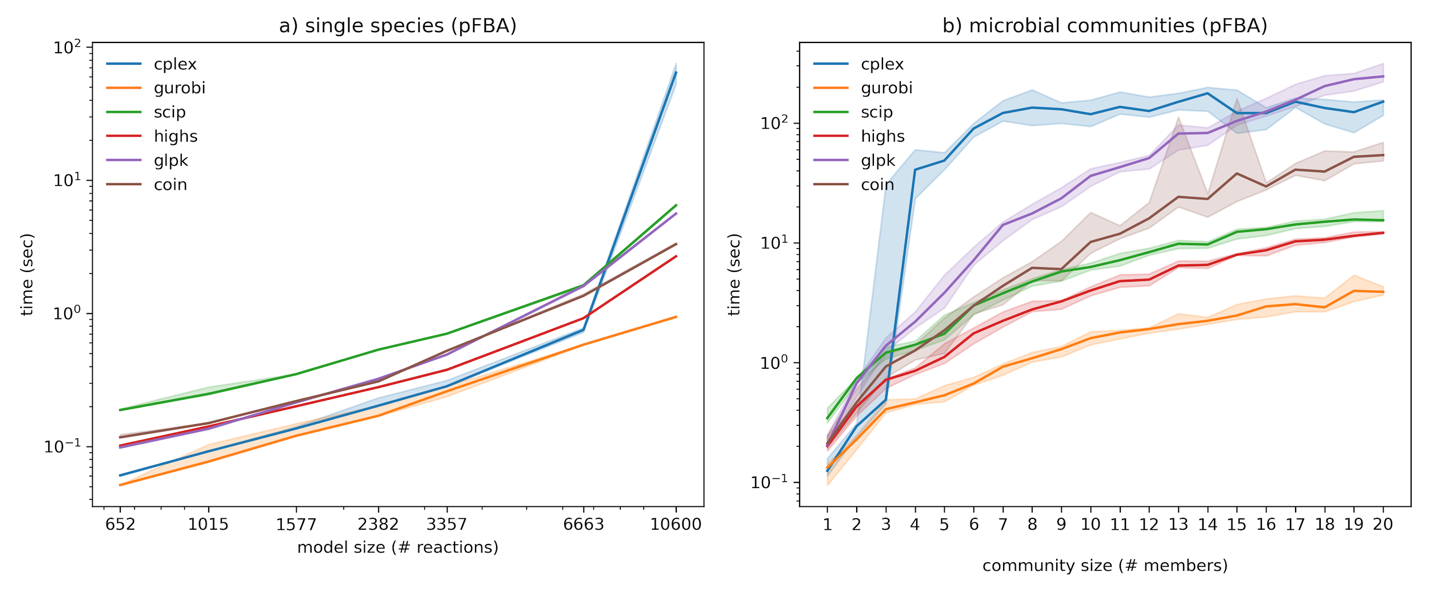


**Supplementary Figure 1:** Benchmarking results for LP formulations. a) Single species pFBA. The horizontal axis (log-scale) represents the number of reactions in each model. b) Simulation of microbial communities (using pFBA). In both panels, the lines represent the median of 10 simulations and the error bands represent the interquartile range.
