# Supplementary material for: A benchmark of optimization solvers for genome-scale metabolic modeling of organisms and communities
Source: mSystems. 2024 Jan 22;9(2):e00833-23. doi: 10.1128/msystems.00833-23 (PMC10878033; doi:10.1128/msystems.00833-23)
Supplement: Fig. S2 — Memory benchmark. [file msystems.00833-23-s0002.docx]

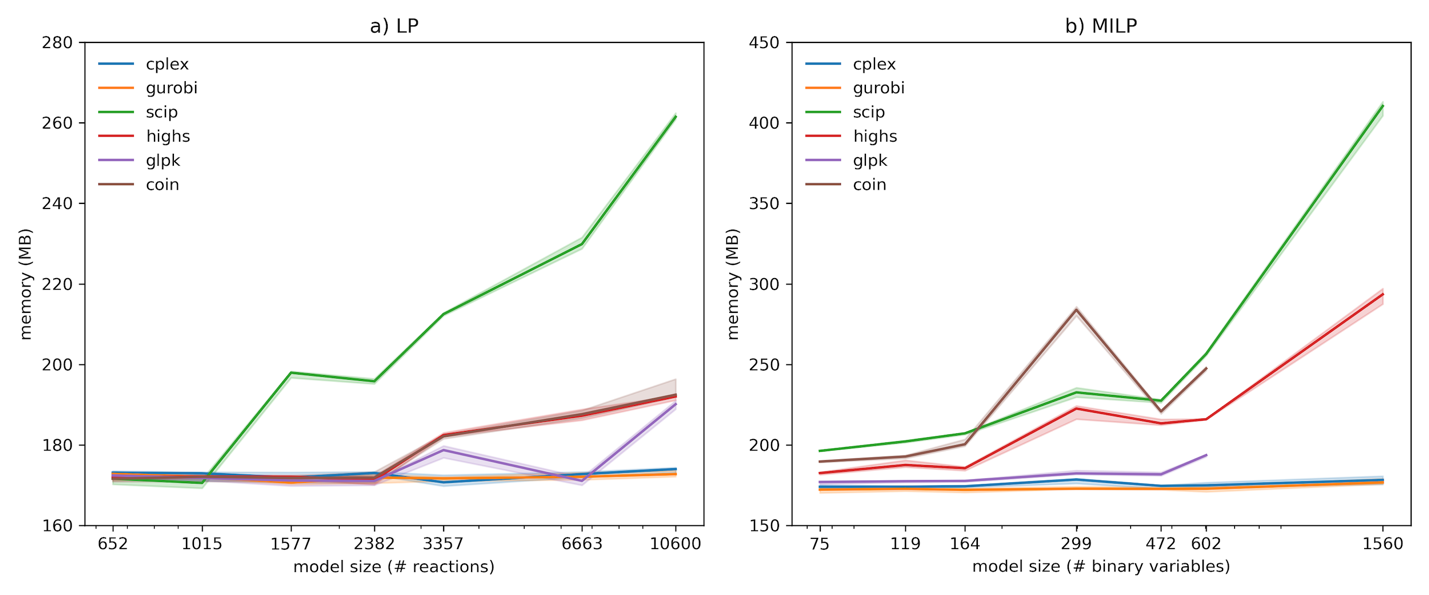


**Supplementary Figure 2:** Benchmarking results for total memory usage. The data shown represents the maximum memory usage detected in each test (including the main process and child processes, measured using Python’s memory profiler). In both panels, the lines represent the median of 10 simulations and the error bands represent the interquartile range.
